# Identification and Quality Evaluation of Raw and Processed *Asarum* Species Using Microscopy, DNA Barcoding, and Gas Chromatography-Mass Spectrometry

**DOI:** 10.1155/2020/2690238

**Published:** 2020-04-13

**Authors:** Guangzhe Yao, Wenjuan Ma, Xuhua Huang, Qi Jia, Jiayuan Shen, Yanxu Chang, Huizi Ouyang, Jun He

**Affiliations:** ^1^First Teaching Hospital of Tianjin University of Traditional Chinese Medicine, Tianjin 300193, China; ^2^Tianjin State Key Laboratory of Modern Chinese Medicine, Tianjin University of Traditional Chinese Medicine, Tianjin.301617, China

## Abstract

*Asarum* (Aristolochiaceae) is one of the common herbs used to relieve exterior syndromes. Some volatile components of *Asarum* which have toxic effect may cause adverse reactions such as headache, general tension, unconsciousness, and respiratory paralysis. Therefore, *Asarum* is normally processed to reduce such toxicity and adverse effects. The bioactive ingredients contained in different *Asarum* herbs vary significantly; this variation may be attributed to their differences in species, origins, or processing methods. In this study, 16 batches of *Asarum* herbs were collected, and their species were identified using DNA barcoding, which is a method for distinguishing plant species, coupled with microscopy. A gas chromatography-mass spectrometry (GC-MS) method for simultaneous determination of 10 compounds was established to evaluate the contents of raw and processed *Asarum* herbs. Multivariate analysis was then applied to compare different batches of herbs based on the GC-MS data. DNA barcoding identified the herbs as being derived from four sources, and herbs from different origins showed different microscopic features. The results demonstrated that most of the samples were clearly clustered into distinct groups that corresponded to species types. All raw and processed samples were classified by partial least squares discriminant analysis (PLS-DA) based on the 10 analyzed compounds. The findings suggested that safrole and methyleugenol with a variable importance in the project (VIP) > 1 are unique compounds that can be used to differentiate between *Asarum* species. Safrole, methyleugenol, and 2,6,6-trimethylcyclohepta-2,4-dien-1-one were identified as significant constituents, the presence of which can be used to differentiate between raw and processed *Asarum* samples. These results indicate that species and processing methods show important effects on the composition of *Asarum* herbs.

## 1. Introduction

According to the *Chinese Pharmacopoeia* (2015 edition), *Asarum* herbs originated from the dry roots and rhizomes of plants in the family Aristolochiaceae, which includes *Asarum heterotropoides* Fr. Schmidt var. *mandshuricum* (Maxim.) Kitag., *Asarum sieboldii* Miq. var. *seoulense* Nakai, and *Asarum sieboldii* Miq [1]. *Asarum maximum* Hemsl. is used as a succedaneum in some regions of China for the treatment of various diseases [2]. The use of the herb was first reported in the monograph *Shennong Compendium of Materia Medica* (*Shennong Bencaojing*) compiled during the Eastern Han dynasty (25–220 AD) [3]. In general, methyleugenol, safrole, and other volatile components (estragole, cineole, and *α*-terpineol, among others) are considered to be the major bioactive components of *Asarum* herbs [4]. Studies have shown that *Asarum* shows antiseptic properties and can also be used to treat cough, dyspnea, headache, rheumatic arthralgia, sinusitis, and toothache [1, 5–7].

The quality of medicinal plants is dependent on genetic and environmental factors; even plants of the same species can have a different chemical composition and function if they are of different geographical origin [8]. *Asarum* is cultivated in various regions of China including Liaoning, Shanxi, and Jilin, which makes the identification of this herb using traditional methods challenging [9]. Characteristic based, microscopic identification, and physicochemical identification are common methods used to identify the constituents of traditional Chinese medicines (TCMs) [10, 11]. However, it is difficult to characterize medicinal materials from multiple sources, especially when the medicines are in the form of a powder. DNA barcoding, a simple operating system, can enable the accurate and rapid identification of raw plants, cells, tissues, decoction products, and powdered medicinal materials and has the benefits of good accuracy and high repeatability [12–16].

In TCM theory, processing (plain-frying or stir-frying until brown, steaming, braising, or stir-frying with wine) is necessary to promote the therapeutic effects and/or reduce the side effects of herbs by decreasing their levels of toxic constituents [17–19]. Chemical reactions may occur during the processing of TCMs, such as hydrolysis reaction, oxidation reaction, displacement reaction, isomerization reaction, and decomposition reaction. For example, after processing *Pinellia ternata* with ginger water, the gingerols in the ginger exert antagonistic effects on the stimulatory toxicity of the *Pinellia* toxic needle crystal [20]. Research has indicated that the compositions of raw and processed *Asarum* herbs are distinct from each other [21]. The combination of mass spectrometry and PLS-DA can provide a comprehensive and multivariate description of the chemical composition of herbal medicines. However, few studies have reported these methods in the multicomponent analysis of *Asarum* or in the comparison of the chemical components that differ between the raw and processed herbs. Therefore, a reliable and comprehensive analytical method is needed to determine several components simultaneously, thus enabling the evaluation of quality in raw and processed samples of *Asarum*.

In our study, microscopic techniques and DNA barcoding were applied to identify the features of and species contained in 16 batches of *Asarum* herbs. Ten volatile components, namely, (1R)-(+)-alpha-pinene, (−)-*β*-pinene, (+)-car-3-ene, cineole, (−)-borneol, safrole, estragole, *α*-terpineol, methyleugenol, and 2,6,6-trimethylcyclohepta-2,4-dien-1-one, were simultaneously evaluated using gas chromatography-mass spectrometry (GC-MS), and the components of raw and processed *Asarum* collected from different areas were analyzed by PLS-DA. This study provides a new point of view on the genetic basis and classification of *Asarum*. Meanwhile, it highlights the influence of processing on the content of volatile components and this information will be useful in the evaluation and differentiation of raw and processed *Asarum* herbs.

## 2. Materials and Methods

### 2.1. Chemicals

Reference standards of (1R)-(+)-alpha-pinene, (+)-car-3-ene, cineole, (−)-*β*-pinene, (−)-borneol, 2,6,6-trimethylcyclohepta-2,4-dien-1-one, *α*-terpineol, estragole, safrole, and methyleugenol were purchased from Desite Biotech Co., Ltd. N-Hexane was provided by Kangkede Technology Co., Ltd. Glycerol was obtained from Tianjin Fengchuan Chemical Reagent Co., Ltd. Chloral hydrate was purchased from Tianjin Kemiou Chemical Reagent Co., Ltd. The plant genomic DNA kit was provided by Tiangen Biotech (Beijing) Co., Ltd. Deionized water was prepared using a Milli-Q water purification system.

### 2.2. Plant Material

*Asarum* herbs from 16 areas were collected from different provinces of China and identified by professor Jun He of the Tianjin State Key Laboratory of Modern Chinese Medicine. *Asarum* was deposited in the TCM library, Tianjin University of Traditional Chinese Medicine, Tianjin, China. The origins of the samples are shown in Table 1.

### 2.3. Microscopic Identification of *Asarum*

The species of *Asarum* were identified according to the root appearance and microscopic features. After screening with a no. 4 sieve, each dried sample was ground into a powder using an electric grinder. Samples were placed on slides and permeated twice with 1–3 drops of chloral hydrate and then sealed with diluted glycerol and a coverslip. Images of *Asarum* powder samples observed with a light microscope under 10x magnification were taken with a digital camera.

### 2.4. DNA Barcoding Analyses

Approximately, 30 mg of dried powder from each sample (roots and rhizomes) was accurately weighed and frozen with liquid nitrogen before undergoing further grinding. The total genomic DNA was isolated from the material using the Plant Genomic DNA Kit DP305 (TIANGEN), according to the manufacturer's instructions, and stored at −20°C.

The primers used to amplify DNA at the ITS2 region were 5′-AGAAGTCGTAACAAGGTTTCCGTAGG-3′ (forward) and 5′-TCCTCCTCCGCTTATTGATATGC-3′ (reverse) [22, 23]. PCR amplification was performed in a 25-*μ*L reaction tube with 2 *μ*L DNA template (10 *μ*M), 12.5 *μ*L 2 × Gflex PCR buffer (Takara), 0.5 *μ*L of each primer, 0.5 *μ*L Tks Gflex DNA polymerase (1.25 units/*μ*L, Takara), and 9 *μ*L ddH_2_O. The PCR program comprised an initial denaturation step at 98°C for 1 min, followed by 40 cycles of denaturation at 98°C for 10 s, annealing at 56.5°C for 15 s, and elongation at 68°C for 30 s, with a final extension step at 68°C for 5 min. The amplified products were detected on 2% agarose gels.

The PCR products were sequenced based on Sanger sequencing technology by the sequencing company (Gitech Solutions Co., Limited). The obtained sequences were searched against the GenBank database using BLAST to determine the species of each sample [24, 25].

### 2.5. Quantification Using GC-MS

#### 2.5.1. Processing Methods of *Asarum*

The raw *Asarum* samples were cut into segments and stir-fried in a metallic pan at 120–140°C over a medium flame, for about 15 min or until the exterior of the sample turns brown colour, with scorched spots.

#### 2.5.2. Preparation of Samples

*Asarum* samples (roots and rhizomes) were dried and grounded into powder. The powder of raw and processed *Asarum* samples was accurately weighed (50.0 g) and subjected to hydrodistillation for 6 h at 100 volt by an essential oil extractor. The essential oil was collected after cooling to room temperature. The essential oil obtained was stored in refrigeration at 4°C. A 5 *μ*L sample of volatile oil (raw or processed) was accurately measured and dissolved in 50 mL of N-hexane.

Ten reference standards: (1R)-(+)-alpha-pinene, (+)-car-3-ene, cineole, *α*-terpineol, (−)-*β*-pinene, (−)-borneol, 2,6,6-trimethylcyclohepta-2,4-dien-1-one, estragole, safrole, and methyleugenol were dissolved in N-hexane at a final concentration of 1 mg/mL as stock solutions, respectively. Working standard solutions were further obtained by diluting the above stock solutions in appropriate amounts.

#### 2.5.3. Gas Chromatographic and Mass Spectrometry Conditions

Automated analysis was performed on a Shimadzu GC-MS QP-2010 Ultrasystem equipped with an AOC-20i autosampler. Chromatographic separations were conducted on a DB-17 capillary column (30 m × 0.25 mm × 0.25 µm film thickness). The column temperature was programmed as follows: 3 min at 40°C, 6°C/min to 106°C, 3°C/min to 142.6°C, 142.6°C for 1 min, 6°C/min to 180°C, and finally 4°C/min to 200°C. High-purity helium was used as the carrier gas at a flow rate of 1.3 mL/min. Split injection was set at a split ratio of 50 : 1, and the injection temperature was 250°C.

The optimized conditions for the MS detector were as follows: ionization source temperature, 230°C; ionization energy, 70 eV; interface temperature, 250°C; full scan range, *m/z* 30–600; and scan rate, 0.30 s per scan. The MS data were acquired in the electron-impact mode. Compounds were identified by the standard substance and NIST08 Mass Spectral Library. Components were quantified by the external standard curve method [7, 26].

#### 2.5.4. GC-MS Method Validation

The linearity of the assay for the test compounds was assessed by least square linear regression of the analyte-to-standard peak area ratio (*y*) versus the normalized standard concentration (*x*). Lower limit of quantification (LLOQ) for each sample was defined based on the concentrations that generated peaks with signal-to-noise values (*S*/*N*) of 10. For precision, the method was evaluated by intraday and interday variability. RSDs were calculated as the measure of precision. In the repeatability examination, six replicates of the samples from the same batch were extracted and analyzed. To evaluate the stability of the analytes, sample solutions were stored at room temperature and then analyzed by replicate injections at 0, 2, 4, 8, 12, and 24 h. RSDs were used to assess stability. The recovery was evaluated by adding known amounts of 10 standard solutions to samples, and these were used to further investigate the accuracy of the method. Recovery was calculated using the following formula: recovery (%) = (amount found − original amount)/amount spiked × 100%.

### 2.6. Statistical Analysis

Differences between the *Asarum* samples were analyzed by PLS-DA. This method establishes the regression relationship between the matrixes, so as to get a better regression prediction result. When a supervised pattern recognition method is employed, the sample data are divided into training and validation sets. The classification model is obtained using the training set, while the accuracy of the prediction is verified using the validation set. In this research, the GC-MS method was applied to analyze the *Asarum* samples including batches of raw and processed *Asarum* samples. A total of 10 volatile compounds were used to evaluate the differences in the above samples. Statistical analyses were carried out using SIMCA-P 14.1 software. The statistical fitness of the model was evaluated by *R*^2^*X*, *R*^2^*Y*, and *Q*^2^.

## 3. Results and Discussion

**Figure 1:**
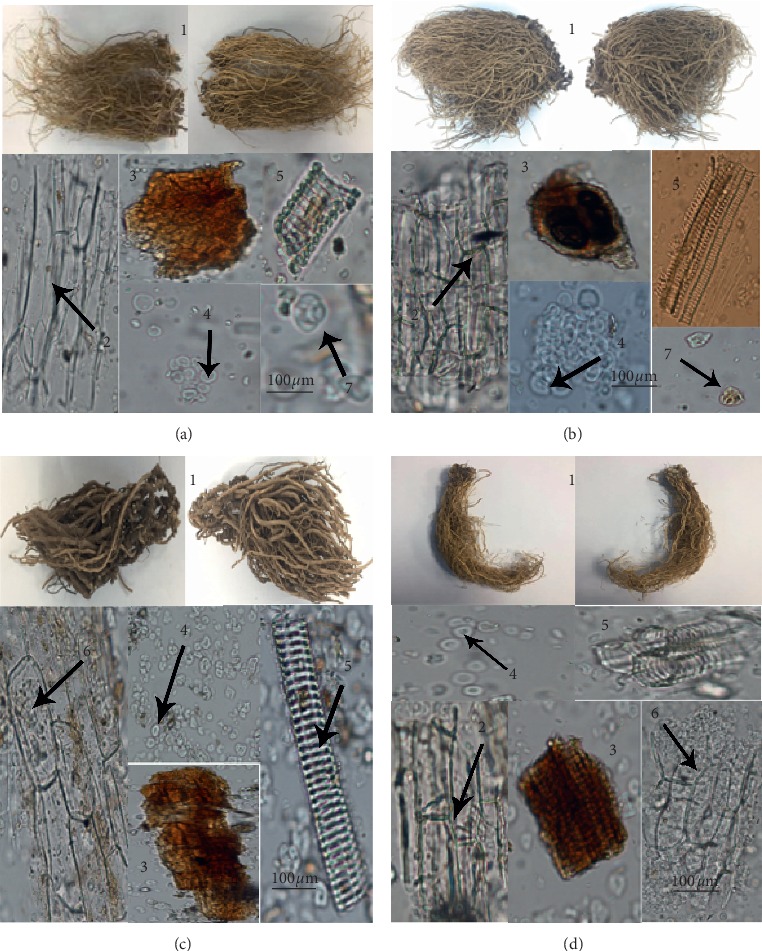
The appearance and microscopic features of *Asarum* samples: (a) *Asarum heterotropoides* Fr. Schmidt var. *mandshuricum* (Maxim.) Kitag.; (b) *Asarum sieboldii* Miq.; (c) *Asarum maximum* Hemsl.; (d) *Asarum sieboldii* Miq. var. *seoulense* Nakai. (1) root; (2) root surface cells; (3) oil cells; (4) starch grain; (5) vessel; (6) secretory cell; and (7) stone cell.

Distinct microscopic features were observed in the upper epidermis, oil cells, starch granules, vessels, and secretory cells. The microscopic appearance and features of *Asarum* are shown in Figure 1. *Asarum heterotropoides* Fr. Schmidt var. *mandshuricum* (Maxim.) Kitag. roots are slender, the surface cells are rectangular and slightly wavy, and they have scalariform vessels and relatively incompact oil cells, with a small number of stone cells and an abundance of starch granules. *Asarum sieboldii* Miq. has long roots, the surface cells are rectangular, and it has scalariform vessels, a small number of stone cells, and an abundance of starch granules. *Asarum sieboldii* Miq. var. *seoulense* Nakai has long, fibrous roots and an abundance of starch granules. *Asarum maximum* Hemsl. has horizontal rhizomes, and it has an abundance of starch granules, but, in our observations, no stone cells. The result demonstrated that the presence or absence of stone cells can be used as a distinguishing feature to differentiate *Asarum heterotropoides* Fr. Schmidt var. *mandshuricum* (Maxim.) Kitag. and *Asarum sieboldii* Miq. from *Asarum maximum* Hemsl. and *Asarum sieboldii* Miq. var. *seoulense* Nakai. The details are provided in Table 1.

After obtaining the results of paired-end sequencing, we use the *de novo* function in the CLC Genomics Work bench to splice the paired-end sequencing. The paired-end reads were merged with Geneious v8.0.4. For the consistence sequence, ITSx v1.1 is used to cut out the ITS2 sequence and modify the degenerate base (when there are low-quality sequence and high-quality sequence nucleotides in the same site, we prefer high-quality sequence nucleotides) [27]. The sequence alignment, which is given in the Supplementary Materials (available here), was obtained by aligning the sequences of different products using MAFFT v7 [28].

**Figure 2:**
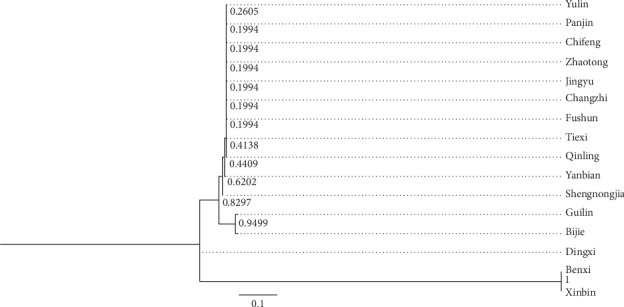
Neighbor-joining (NJ) phylogenetic trees of different *Asarum* species based on ITS2 sequences.

The sequence alignment was compared with the GenBank database, which used the Basic Local Alignment Search Tool (BLAST) to determine the species contained in each sample [29]. The results indicated that the *Asarum* herbs were derived from four species including *Asarum* heterotropoides Fr. Schmidt var. mandshuricum (Maxim.) Kitag., *Asarum* sieboldii Miq., *Asarum* sieboldii Miq. Var. *seoulense* Nakai, and *Asarum maximum* Hemsl (Table 2). We also used the sequence alignment to construct the neighbor-joining (NJ) phylogenetic tree by NJ tools with MEGA-X molecular evolutionary genetic analysis software (see Figure 2). The results demonstrated that *Asarum* samples from four species formed a stable branch. The type of compounds were similar in closely related species. The branches of *Asarum* sieboldii Miq. were dispersed, which was probably related to the number of samples. By comparative analysis of the ITS2 sequence, we found that numerous transformation/transversion and insertion/deletion phenomena had occurred in the ITS2 sequence, and the information loci were abundant, which could be used for molecular identification of these samples.

**Figure 3:**
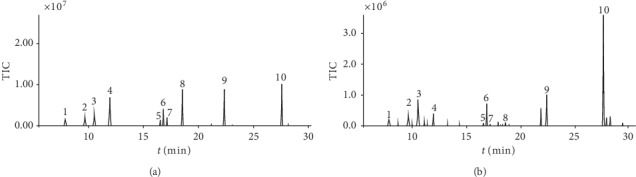
Full-scan monitoring chromatograms of (1R)-(+)-alpha-pinene (1), (−)-*β*-pinene (2), (+)-car-3-ene (3), cineole (4), (−)-borneol (5), 2,6,6-trimethylcyclohepta-2,4-dien-1-one (6), *α*-terpineol (7), estragole (8), safrole (9), and methyleugenol (10): (a) standard solution; (b) *Asarum* sample.

The linear calibration curves of peak areas (*y*) versus concentrations (*x*) were plotted for the 10 volatile compounds. The regression coefficients (*r*^2^) were >0.994 for the 10 compounds, indicating good linearity within a relatively wide range of concentrations. For precision, the relative standard deviations (RSD) for the contents of the 10 characteristic components ranged from 1.8 to 6.1%. In a test of repeatability, the RSD values for all target analytes ranged from 2.3 to 8.9%. The results indicated that the method is precise and repeatable. Stability, measured as RSD, was in the range 2.2–6.6%, indicating that the samples were stable for 24 h. The recovery of each analyte ranged from 89.2 to 113.3%. These results indicated that the efficiency of sample preparation was acceptable. The full-scan monitoring chromatograms of the analytes are shown in Figure 3. The validation data shown in Table 3 are considered to be satisfactory for the subsequent analysis of all samples.

**Figure 4:**
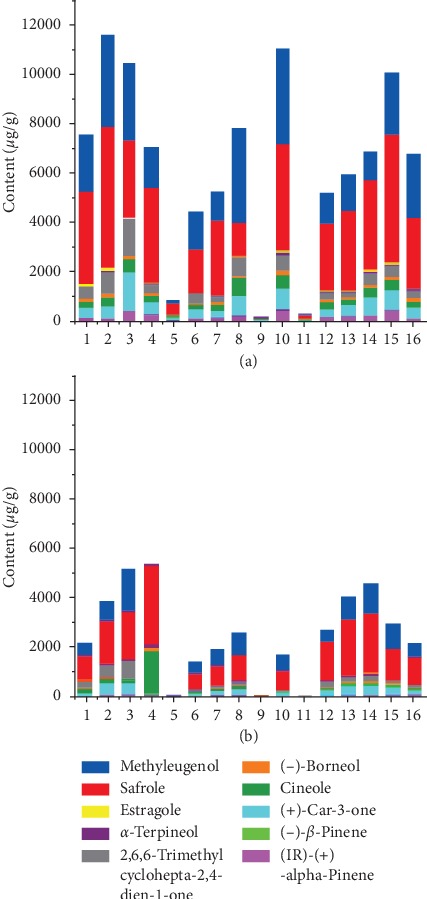
The total contents of 10 volatile components in different batches of raw (a) and processed (b) *Asarum* samples (*μ*g/g) (1, Jingyu; 2, Yanbian; 3, Fushun; 4, Zhaotong; 5, Guilin; 6, Shennongjia; 7, Xinbin; 8, Tiexi; 9, Dingxi; 10, Changzhi; 11, Bijie; 12, Benxi; 13, Panjin; 14, Chifeng; 15, Qinling; 16, Yulin).

The validated method was applied to the analysis of 16 batches of raw and processed *Asarum* samples. Ten bioactive compounds of *Asarum* were quantified using the external standard method based on their respective calibration curves. The contents of the 10 compounds in raw and processed *Asarum* samples are listed in Table 4. There were differences in the contents of analytes between raw and processed samples. As illustrated in Figure 4, there were obvious differences in the total contents of 10 components. For example, the total contents of 10 ingredients in processed *Asarum* were significantly decreased when compared with raw *Asarum*.

**Figure 5:**
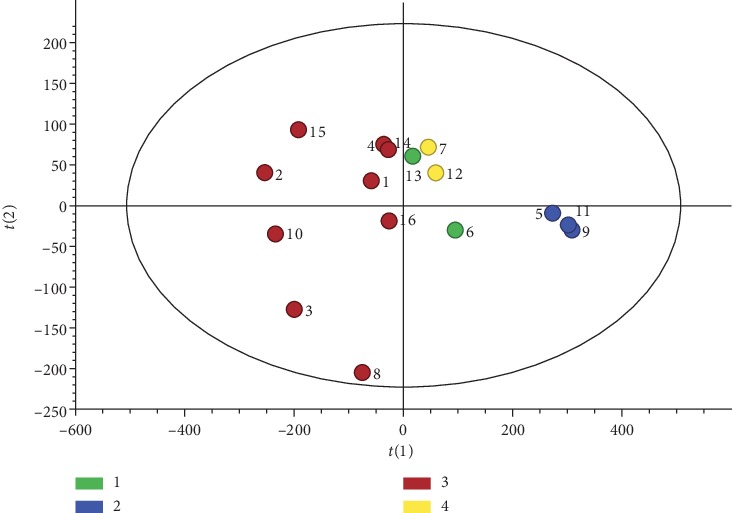
PLS-DA score scatter plot for samples collected from different species: (1) *Asarum sieboldii* Miq.; (2) *Asarum maximum* Hemsl.; (3) *Asarum heterotropoides* Fr. Schmidt var. *mandshuricum* (Maxim.) Kitag.; and (4) *Asarum sieboldii* Miq. var. *seoulense* Nakai (*R*^2^*X* = 0.895, *R*^2^*Y* = 0.361, *Q*^2^ = 0.246) (1, Jingyu; 2, Yanbian; 3, Fushun; 4, Zhaotong; 5, Guilin; 6, Shennongjia; 7, Xinbin; 8, Tiexi; 9, Dingxi; 10, Changzhi; 11, Bijie; 12, Benxi; 13, Panjin; 14, Chifeng; 15, Qinling; 16, Yulin).

**Figure 6:**
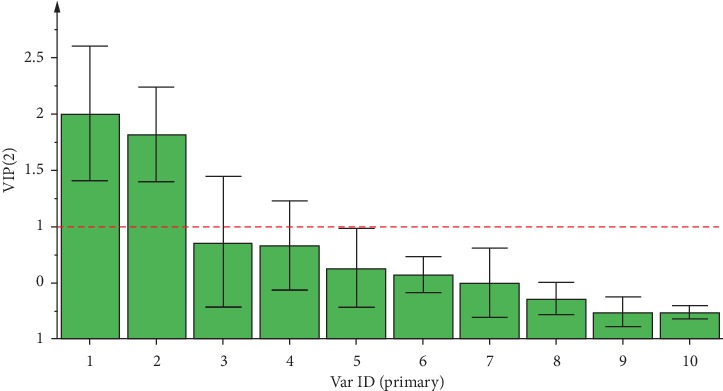
VIP score plot for PLS-DA of *Asarum* samples collected from different species: (1) safrole; (2) methyleugenol; (3) (+)-car-3-ene; (4) 2,6,6-trimethylcyclohepta-2,4-dien-1-one; (5) cineole; (6) (−)-*β*-pinene; (7) (1R)-(+)-alpha-pinene; (8) (−)-borneol; (9) *α*-terpineol; and (10) estragole.

Most samples were clearly clustered into distinct groups corresponding to their species types (see Figure 5). The samples of *Asarum heterotropoides* Fr. Schmidt var. *mandshuricum* (Maxim.) Kitag. and *Asarum sieboldii* Miq. were relatively scattered but *Asarum maximum* Hemsl. samples were more closely clustered together. Compared with *Asarum maximum* Hemsl., *Asarum sieboldii* Miq. and *Asarum sieboldii* Miq. var. *seoulense* Nakai were located closer to *Asarum heterotropoides* Fr. Schmidt var. *mandshuricum* (Maxim.) Kitag. The corresponding score plot combined with VIP values screened out compounds, namely, safrole and methyleugenol for the differentiation of the various *Asarum* samples. The important variables were selected using the criteria that VIP > 1 (see Figure 6). The result of the PLS-DA demonstrated that variations in content differences could be due to different species being present in the *Asarum* samples.

**Figure 7:**
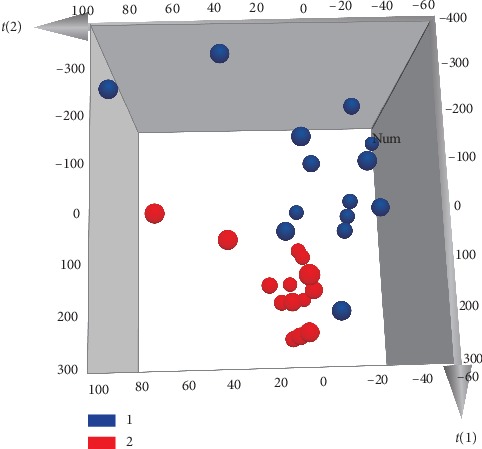
PLS-DA 3D score scatter plot for raw (1) and processed (2) *Asarum* (*R*^2^*X* = 0.861, *R*^2^*Y* = 0.44, *Q*^2^ = 0.344).

**Figure 8:**
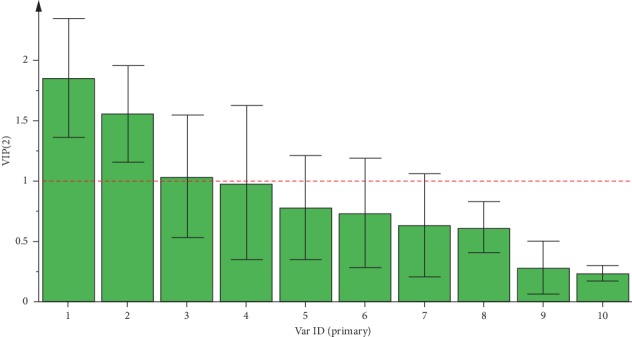
VIP score plot for PLS-DA of raw and processed *Asarum* samples: (1) safrole; (2) methyl eugenol; (3) 2,6,6-trimethylcyclohepta-2,4-dien-1-one; (4) cineole; (5) (+)-car-3-ene; (6) (−)-*β*-pinene; (7) (−)-borneol; (8) (1R)-(+)-alpha-pinene; (9) estragole; and (10) *α*-terpineol.

The GC-MS results were further analyzed by PLS-DA. A three-dimensional (3D) score plot of the PLS-DA was carried out to measure the differences between raw and processed *Asarum* (see Figure 7). Raw *Asarum* samples clustered in a relatively discrete larger region, which was distinct from that of the processed samples. The processed samples were in a small region, which indicates that the processed samples are more stable than those of the raw samples. Constituents with large loading values can be considered as markers, which contributed clearly to the classification of the samples. In the present study, potential bioactive ingredients such as safrole, methyleugenol, and 2,6,6-trimethylcyclohepta-2,4-dien-1-one with VIP >1 were identified as compounds that could be used to differentiate between the raw and processed *Asarum* samples (see Figure 8). The results showed that the chemical composition of *Asarum* samples was altered after processing. The above components contributed greatly to sample classification and may be the material basis for the change of clinical efficacy associated with herb processing.

## 4. Conclusions

In this study, we carried out the character and microscopic identification of *Asarum* from different species. Microscopic observations of *Asarum* powder samples indicated that a variety of features appeared in different kinds of *Asarum* samples. As there were many identical cell structures across all samples, a single differential feature could not distinguish the different species apart. Molecular identification technology was then used to identify the above samples, and four species of *Asarum* were clearly identified from the 16 batches of *Asarum* samples. Such molecular identification technology makes up for the deficiency of character and microscopic identification.

In addition, a GC-MS method was developed and validated for the simultaneous determination of 10 active components in *Asarum* samples, and the contents of 16 batches of *Asarum* samples were investigated by this method, which confirmed the results of the molecular identification. In light of the quantification data of 10 active constituents, PLS-DA analysis was carried out to discriminate and predict *Asarum* samples of different species and processing methods. Results indicated that raw *Asarum* samples from different sources in 16 regions could be clearly classified into four subclusters by PLS-DA, which verified the results of molecular identification. Two volatile oils (safrole and methyleugenol) with a VIP > 1 were identified, based on PLS-DA, as key compounds that can be helpful to distinguish the different species apart. Furthermore, the PLS-DA model showed that raw and processed *Asarum* were clustered in two different areas, respectively. Safrole, methyleugenol, and 2,6,6-trimethylcyclohepta-2,4-dien-1-one were significant index constituents to differentiate *Asarum* samples of different processing methods. The above methods, combining microscopic identification, DNA barcoding, and GC-MS, were established for the identification of raw *Asarum* and its processed products, which provided the basis for the quality evaluation and species differences of *Asarum*.

## Figures and Tables

**Table 1:** Detailed information of *Asarum* from 16 regions.

Batch	Origin	Lot number	Appearance features	Power characteristics
1	Jingyu	20180817	Roots long, multibranch	The surface cells rectangular and slightly wavy, scalariform vessel, oil cells relatively incompact, small number of stone cells, more starches
2	Yanbian	20180820	Roots long, multibranch	The surface cells rectangular and slightly wavy, scalariform vessel, oil cells relatively incompact, small number of stone cells, more starches
3	Fushun	20180817	Roots long, multibranch	The surface cells rectangular and slightly wavy, scalariform vessel, oil cells relatively incompact, small number of stone cells, more starches
4	Zhaotong	20180915	Roots long, multibranch	The surface cells rectangular and slightly wavy, scalariform vessel, oil cells relatively incompact, small number of stone cells, more starches
5	Guilin	20180807	Roots relatively long and thick,	More starches and wood fiber, stone cells none.
6	Shennongjia	20180620	Roots long, multibranch	The surface cells rectangular, scalariform vessel, a small number of stone cells, and more starches.
7	Xinbin	20180802	Roots long, multibranch	The surface cells rectangular, scalariform vessel, and more starches.
8	Tiexi	20180904	Roots long, multibranch	The surface cells rectangular and slightly wavy, scalariform vessel, oil cells relatively incompact, small number of stone cells, more starches
9	Dingxi	20181016	Roots relatively long and thick	More starches and wood fiber, stone cells none.
10	Changzhi	20180306	Roots long and thin multibranch	The surface cells rectangular and slightly wavy, scalariform vessel, oil cells relatively incompact, small number of stone cells, more starches
11	Bijie	20180818	Roots relatively long and thick, less branch	More starches and wood fiber, stone cells none.
12	Benxi	20180818	Roots long and thin, more fibrous roots	The surface cells rectangular, scalariform vessel, and more starches.
13	Panjin	20180811	Roots long, multibranch	The surface cells rectangular, scalariform vessel, a small number of stone cells, and more starches.
14	Chifeng	20181011	Roots long and thin, multibranch	The surface cells rectangular and slightly wavy, scalariform vessel, oil cells relatively incompact, small number of stone cells, more starches
15	Qinling	20180726	Roots long and thin, multibranch	The surface cells rectangular and slightly wavy, scalariform vessel, oil cells relatively incompact, more starches
16	Yulin	20180721	Roots long and thin, multibranch	The surface cells rectangular and slightly wavy, scalariform vessel, oil cells relatively incompact, more starches

**Table 2:** The molecular identification result information.

Batch	Origin	Length (bp)	DNA identification	Reference accession number
1	Jingyu	221	*Asarum heterotropoides* Fr. Schmidt var. *mandshuricum* (maxim.) Kitag.	KX674960.1
2	Yanbian	221	*Asarum heterotropoides* Fr. Schmidt var. *mandshuricum* (maxim.) Kitag.	KX674960.1
3	Fushun	221	*Asarum heterotropoides* Fr. Schmidt var. *mandshuricum* (maxim.) Kitag.	KX674960.1
4	Zhaotong	221	*Asarum heterotropoides* Fr. Schmidt var. *mandshuricum* (maxim.) Kitag.	KX674960.1
5	Guilin	221	*Asarum maximum* Hemsl.	FJ980374.1
6	Shennongjia	221	*Asarum sieboldii* Miq.	MF096022.1
7	Xinbin	221	*Asarum sieboldii* Miq. Var. *seoulense* Nakai	AB247109.1
8	Tiexi	221	*Asarum heterotropoides* Fr. Schmidt var. *mandshuricum* (maxim.) Kitag.	KX674960.1
9	Dingxi	221	*Asarum maximum* Hemsl.	FJ980374.1
10	Changzhi	221	*Asarum heterotropoides* Fr. Schmidt var. *mandshuricum* (maxim.) Kitag.	KX674960.1
11	Bijie	221	*Asarum maximum* Hemsl.	FJ980374.1
12	Benxi	221	*Asarum sieboldii* Miq. Var. *seoulense* Nakai	AB247109.1
13	Panjin	221	*Asarum sieboldii* Miq.	MF096022.1
14	Chifeng	221	*Asarum heterotropoides* Fr. Schmidt var. *mandshuricum* (maxim.) Kitag.	KX674960.1
15	Qinling	221	*Asarum heterotropoides* Fr. Schmidt var. *mandshuricum* (maxim.) Kitag.	KX674960.1
16	Yulin	221	*Asarum heterotropoides* Fr. Schmidt var. *mandshuricum* (maxim.) Kitag.	KX674960.1

**Table 3:** Regression equation, linear range, correlation coefficients (*r*^2^), LLOQ, precision, repeatability, stability, and recovery of 10 investigated analytes (*n* = 6).

Compounds	Regression equation	Linear range (*μ*g/mL)	*r* ^2^	LLOQ (*μ*g/mL)	Precision RSD (%)	Repeatability RSD (%)	Stability RSD (%)	Recovery (%)
(1R)-(+)-alpha-Pinene	*y* = 110696.5*x* − 19785.11	0.08–64	0.999	0.03	3.4	4.5	2.2	94.8
(−)-*β*-Pinene	*y* = 103513.6*x* − 18753.84	0.1–80	0.999	0.05	4.0	4.9	2.7	105.7
(+)-Car-3-ene	*y* = 81430.83*x* + 6001.557	0.1–80	0.999	0.02	4.2	4.4	2.5	102.6
Cineole	*y* = 31324.58*x* + 51269.66	0.25–200	0.998	0.03	4.3	8.9	4.1	95.5
(−)-Borneol	*y* = 99069.16*x* − 5912.898	0.1–80	0.999	0.02	3.4	6.1	2.6	108.1
2,6,6-Trimethylcyclohepta-2,4-dien-1-one	*y* = 51336.33*x* + 33969.81	0.12–96	0.999	0.02	6.1	4.5	5.4	94.5
*α*-Terpineol	*y* = 61057.91*x* − 10884.64	0.05–40	0.999	0.04	6.0	8.8	6.6	89.2
Estragole	*y* = 49990.04*x* + 171669.0	0.3–240	0.996	0.01	1.8	6.5	5.5	102.3
Safrole	*y* = 35569.62*x* + 225493.1	0.6–480	0.997	0.02	4.0	5.5	3.6	111.3
Methyleugenol	*y* = 51970.14*x* + 466412.8	0.6–480	0.994	0.01	5.1	2.3	5.1	101.2

**Table 4:** The contents of 10 compounds in *Asarum* samples (*μ*g/g).

Compounds	Sort	1	2	3	4	5	6	7	8	9	10	11	12	13	14	15	16
(1R)-(+)-alpha-Pinene	Crude	1679	1337	4330	2958	595	1404	1677	2399	234	4602	193	2009	2187	2676	4751	1707
	Processed	678	1043	1197	836	171	532	1065	918	62	266	31	703	1160	1039	1128	1141
(−)-*β*-Pinene	Crude	2178	1978	5014	2623	837	1646	3611	3345	158	4017	168	1882	3111	3961	5086	2832
	Processed	923	1003	1675	2509	243	599	1366	1056	48	359	28	714	2146	1757	1247	1247
(+)-Car-3-ene	Crude	3600	4834	15849	4931	953	3534	3151	7880	498	8868	371	3058	4539	7276	8053	4230
	Processed	965	4693	4784	387	54	1218	1723	2451	166	1549	34	2378	3570	3247	2874	1864
Cineole	Crude	2516	3669	4920	2883	808	1782	2102	7370	500	5684	511	2766	2135	3828	4103	2382
	Processed	1648	1756	1126	17292	101	397	540	1554	201	416	44	625	988	1463	995	736
(−)-Borneol	Crude	1137	1392	1390	1065	430	643	1103	762	70	1502	136	1080	946	1160	1263	839
	Processed	382	395	333	1244	130	197	315	282	42	94	22	378	540	631	310	288
2,6,6-Trimethyl-cyclohepta-2,4-dien-1-one	Crude	5070	8988	15335	3230	376	3660	2200	7004	—	6232	51	3132	2253	4899	4520	3484
	Processed	2987	5340	7440	—	122	1036	877	1395	—	652	4	2480	2074	2554	1414	977
*α*-Terpineol	Crude	490	551	576	597	98	342	425	474	37	1005	49	405	452	628	531	349
	Processed	153	284	168	1572	41	169	173	227	27	154	9	150	387	436	159	204
Estragole	Crude	586	1216	—	195	—	375	59	568	—	992	—	320	274	618	581	408
	Processed	262	—	—	—	—	—	—	55	—	—	—	126	201	374	—	—
Safrole	Crude	37390	57103	31628	38732	4368	17850	30086	13249	112	43367	1216	27204	32387	36568	52336	28725
	Processed	9682	17624	19532	32002	—	5978	8183	10534	109	7845	15	15483	22608	24071	12780	11025
Methyleugenol	Crude	22889	36592	30139	15816	808	14699	11641	38261	103	37887	138	11539	13975	10897	24529	25499
	Processed	4699	7179	17151	212	89	4712	6310	8041	—	5986	18	4843	8785	11538	9449	5122

Annotation: 1, Jingyu; 2, Yanbian; 3, Fushun; 4, Zhaotong; 5, Guilin; 6, Shennongjia; 7, Xinbin; 8. Tiexi; 9. Dingxi; 10, Changzhi; 11, Bijie; 12, Benxi; 13, Panjin; 14, Chifeng; 15, Qinling; 16, Yulin.

## Data Availability

The data used to support the findings of this study are available from the corresponding author upon request.

## Supplementary Materials

Supplementary Materials Alignment sequences of Asarum from 16 regions.
