# Supplementary figures and images for: Identification and Quality Evaluation of Raw and Processed Asarum Species Using Microscopy, DNA Barcoding, and Gas Chromatography-Mass Spectrometry
Source: J Anal Methods Chem. 2020 Apr 13;2020:2690238. doi: 10.1155/2020/2690238 (PMC7174948; doi:10.1155/2020/2690238)

**Supplementary Materials**


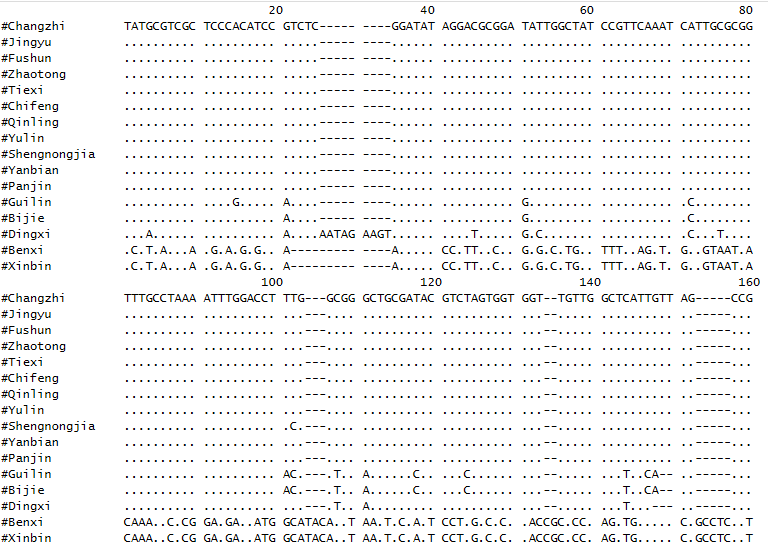


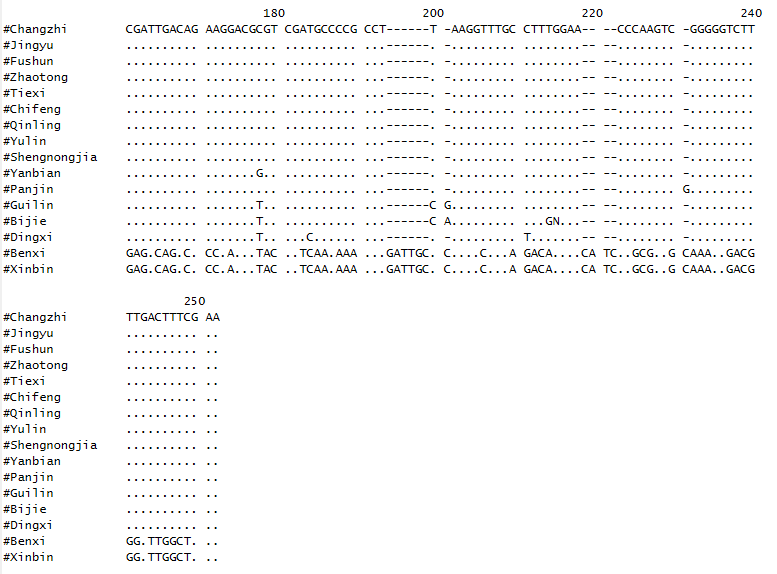


Alignment sequences of *Asarum* from 16 regions

Supplement: Supplementary Materials — Alignment sequences of Asarum from 16 regions. [file 2690238.f1.doc]
